# Correction to “Quantitative Monitoring of Diverse Fish Communities at a Large Scale Combining eDNA Metabarcoding and qPCR”

**DOI:** 10.1111/1755-0998.70193

**Published:** 2026-08-25

Pont, D., P. Meulenbroek, V. Bammer, et al. 2023. “Quantitative Monitoring of Diverse Fish Communities on a Large Scale Combining eDNA Metabarcoding and qPCR.” *Molecular Ecology Resources* 23: 396–409. https://doi.org/10.1111/1755‐0998.13715.

Due to an error in the calculation of teleo‐eDNA concentrations (real time qPCR), all figures expressing the number of teleo‐eDNA copies per litre or per sample must be divided by 100.

The results of the article remain unchanged:

1. The strength of the correlation between the total number of teleo‐eDNA copies per litre and fish abundance/biomass per hectare (conventional fishing) is independent of the units of the two variables.
2. The strength of the correlation between the total number of teleo‐eDNA copies per litre and the mean annual water discharge (logarithmic scale) at the different sites of the Danube and its tributaries is also dimensionless.
3. The non‐linear asymptotic model describing the species–sampling effort relationship (number of species detected vs. teleo‐DNA concentration per sample) is unchanged, as the numerical values of the total number of teleo‐DNA copies per sample have been rescaled to avoid instabilities due to extremely high numerical values. The only modification concerns the unit of the x‐axis of Figure 7: The initial numerical values of the number of DNA copies must be divided by a factor of 10^4^ instead of 10^6^ (see corrected Figure 7).
4. The LOD and LOQ values are not affected by this correction.
5. The corrected predicted value of teleo‐DNA required to detect 95% of the taxonomic richness is then 6,510 DNA copies extracted per sample, considering the model parameters defined at the population level (see the Results section and Abstract).

Figures 4, 5 and 7 are corrected (modifications of the axis numerical values and axis labels when necessary). Figures 1, 2, 3 and 6 are unchanged.

**FIGURE 4:**
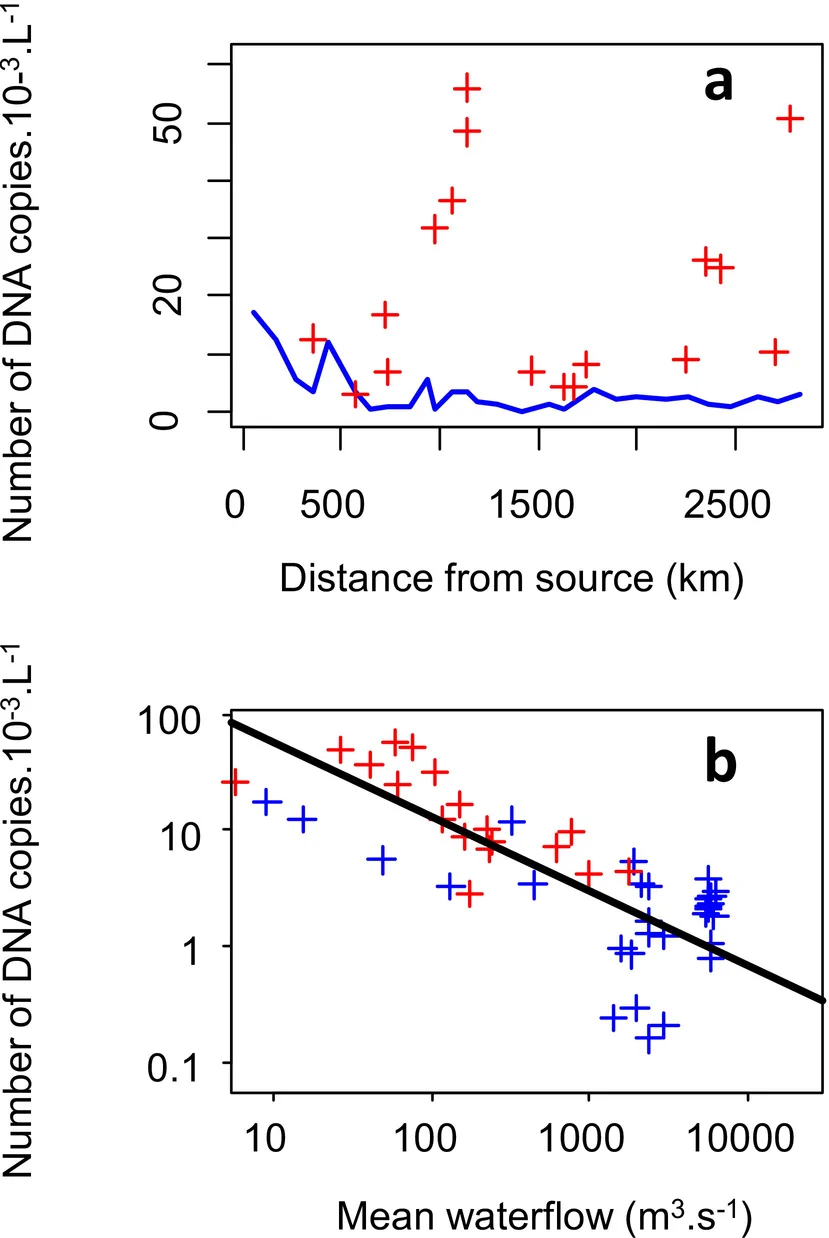
Between‐site variability of teleo‐DNA concentrations in the Danube (blue line) and the tributaries (red cross) (a). Relationship between teleo‐DNA concentrations and mean annual water flow (log scales) at the different sites from the Danube (blue) and the tributaries (red) (b).

**FIGURE 5:**
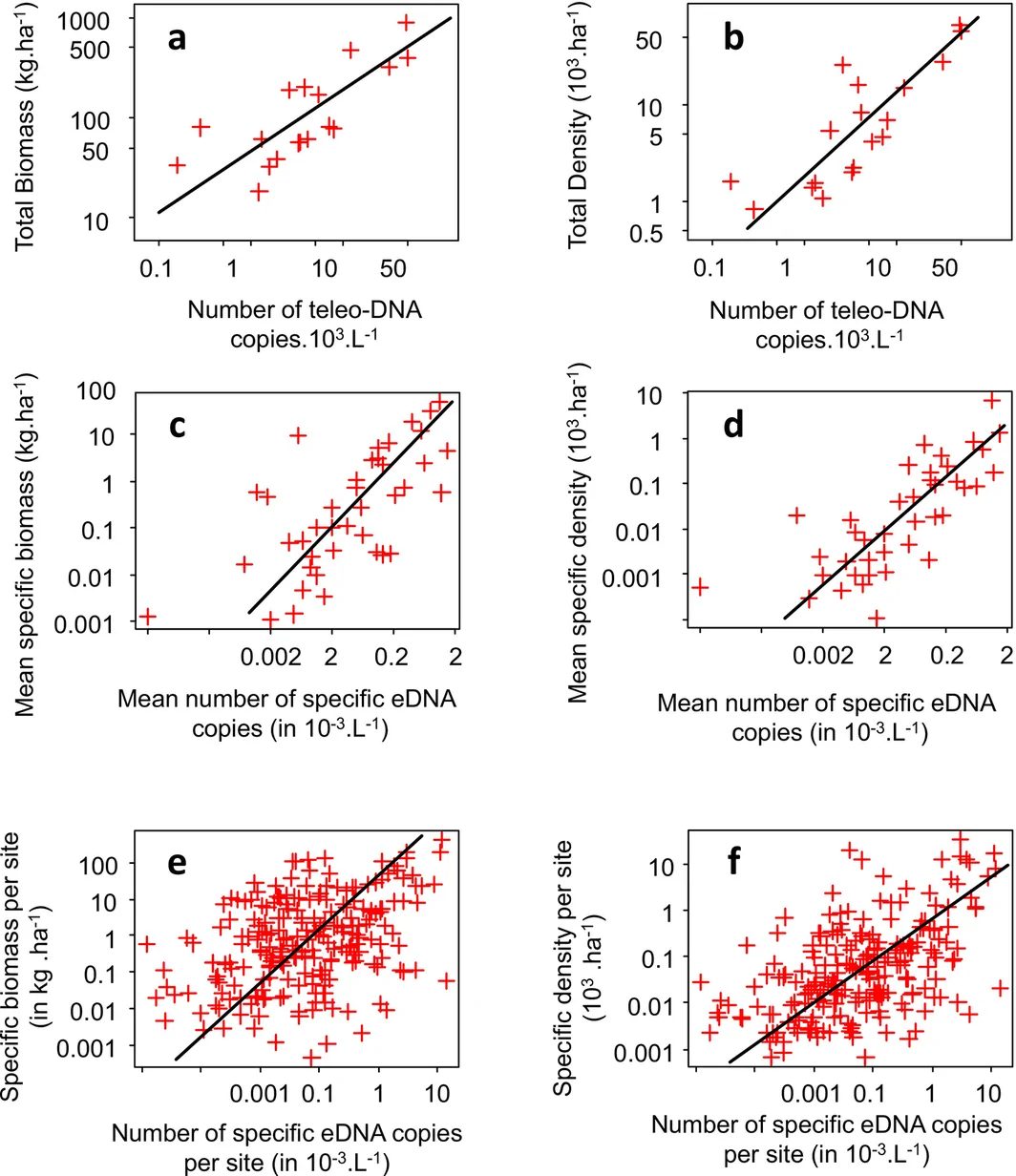
Comparison between eDNA and traditional electrofishing methods (TEF) at the 18 common sites sampled by both methods. Regressions (type II) of teleo‐eDNA concentration (mitochondrial DNA copies·10^−3^·L^−1^) on total fish biomass (a) and total fish density (b) per site estimated by TEF. Regressions (type II) of mean species‐specific eDNA concentration on mean biomasses (c) and mean densities (d) obtained by TEF when all sites are combined. Regressions (type II) of species‐specific eDNA concentration per site on species‐specific biomasses (e) and species‐specific densities estimated by TEF per site (f).

**FIGURE 7:**
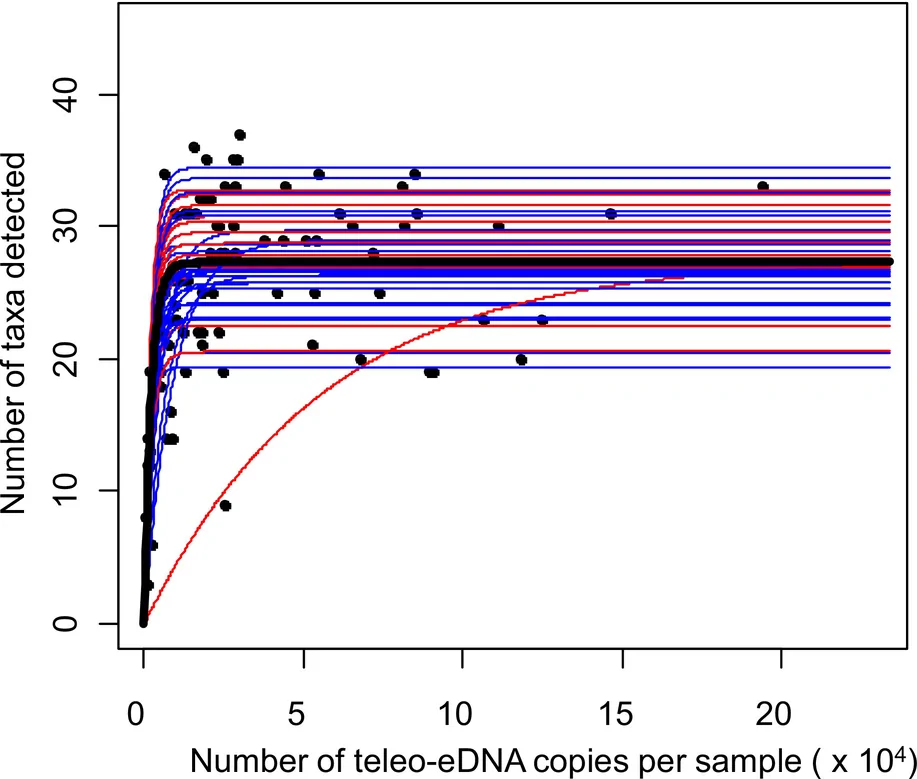
Plot of the number of taxa detected by eDNA against the number of teleo‐DNA copies per sample for the 47 sites. Fitted curves from parameters estimated from a non‐linear mixed model at the population level (black line) and the individual level (red lines: tributaries, blue lines: the Danube River). Longitudinal distribution of species.

We apologize for this error.

